# Assessment of Functional Properties of Wheat–Cassava Composite Flour

**DOI:** 10.3390/foods12193585

**Published:** 2023-09-27

**Authors:** Mingjuan Li, Yayuan Zhang, Xiangrong You, Ying Wang, Kui Zhou, Ping Wei, Linyan Wei

**Affiliations:** 1 Agro-Food Science and Technology Research Institute, Guangxi Academy of Agricultural Sciences, Nanning 530007, China; limingjuan230@163.com (M.L.);; 2 Guangxi Key Laboratory of Fruits and Vegetables Storage-Processing Technology, Nanning 530007, China

**Keywords:** cassava flour (CF), wheat–cassava composite flour (W-CF), dough, functional properties, physicochemical qualities

## Abstract

Cassava flour (CF) was used as a raw material to replace wheat flour (WF) at levels of 0% (control), 10%, 20%, 30%, 40%, and 50% to prepare wheat–cassava composite flour (W-CF) and dough. The effects of different CF substituting levels on the functional properties of the W-CF and dough were investigated. The results show that an increase in CF led to a decrease in the moisture, protein, fat, and b* values of W-CF. The crude fiber, ash, starch, L*, a* values, iodine blue value (*IBV*), and swelling power (*SP*) of the composite flour increased gradually. It was found that the water absorption, hardness, and chewiness of the W-CF dough increased with an increase in the CF substitution level. A different trend could be observed with the springiness and cohesiveness of the W-CF dough. The resistance to extension, extensibility, and the extended area of the W-CF dough at all substitution levels was significantly lower than that of the WF dough. The elasticity and cohesiveness of the dough tended to increase for CF content from 10% to 30%, followed by a decrease at a higher replacement. Pearson correlation analysis indicated that the substitution levels of CF had a significant influence on the proximate analysis and functional properties of the W-CF and dough. This study will provide important information on choosing CF substitution levels for different products.

## 1. Introduction

Cassava (*Manihot esculenta* Crantz) is an important tuber crops cultivated in tropical and subtropical regions of the world that has a strong tolerance against soil infertility and water stress [1]. In Africa and South America, cassava is consumed as an important staple food by more than 800 million people [2]. Fresh cassava roots are difficult to store and usually have a short shelf life of only 1~2 days after harvest because of their postharvest physiological deterioration (PPD) [3,4]. Therefore, cassava is generally prepared as flour by peeling, crushing, and drying. The production of cassava flour (CF) can prolong the shelf-life of cassava and further reduce cyanide glucosides content to safe levels [5]. 

Cassava flour has attractive advantages in terms of starch, with low retrogradation, high water-binding ability, and high fiber and mineral content compared with cereal flours. Numerous studies and applications demonstrated the feasibility of partially replacing wheat flour with cassava flour in cakes [6], biscuits [7], bread [8], and noodles [9]. Compared with wheat products, the food produced with cassava flour provided more carbohydrates and low lipid and protein content [7]. The research indicated that the inclusion of cassava flour in bread production led to a less significant glycemic response [10]. However, cassava flour has a negative effect with replacement at higher levels because of the absence of gluten. Previous studies showed that the sensory quality of bread with a substitution level of 10% or 20% is generally acceptable. Increasing the substitution ratio of cassava flour has been shown to result in a decrease in the specific volume and sensory quality of bread [11]. 

The total or partial replacement of wheat flour (WF) with cassava flour can promote the use of cassava in wheat-importing countries and enhance the economic value of cassava. Thus, the aim of this study was to investigate the influence of the partial substitution of wheat flour with cassava flour at the levels of 0~50% on the function properties of composite flours and their doughs. The correlation between CF substitution level, the function properties of W-CF, and dough was also analyzed to provide information for the further development and application of cassava flour in baked food.

## 2. Materials and Methods

### 2.1. Materials

Wheat flour was purchased from a local market, produced by the company Henan Xuejian Industrial Co., Ltd., Luohe, China. The cassavas, namely South China No.9 (SC9), were harvested from the experimental farm at the Guangxi Academy of Agricultural Sciences (Nanning, China). 

### 2.2. Sample Preparation

The freshly harvested cassava tubers were peeled, cleaned, and cut into slices with a thickness of 3~5 mm, and the root slices were dried at 50 °C in a heat air cabinet drier for 10 h. The dried slices were then ground by KC-701 ultrafine grinder (Beijing Kaichuang Tonghe Technology Development Co., Ltd., Beijing, China) until they were all passed through 100 mesh sieves to obtain cassava flour. The resulting cassava flour was collected, packaged, and sealed for further analysis.

Wheat–cassava composited flours were prepared by replacing wheat flour with cassava flour at the levels of 10%, 20%, 30%, 40%, and 50% (dry weight basis, db), respectively. Wheat flour was used as the control. 

The dough was made from 100 g of wheat and wheat–cassava composited flours by a laboratory-type dough kneading machine (Kenwood Chef Major KMC510, Treviso, Italy), while the water addition was calculated according to the following formula so that the water content of W-CF was adjusted to 45%. The mixing time was about 8 min, until a homogeneous mass was obtained. The prepared dough was wrapped with plastic film and placed at 25 ± 1 °C for 30 min. After resting, the dough was divided by hand into rounds with a weight of about 30 g. The water addition formula is as follows:

$$\frac{\mathrm{weight}\;\mathrm{of}\;\mathrm{WF}\times \mathrm{water}\;\mathrm{content}\;\mathrm{of}\;\mathrm{WF}+\mathrm{weight}\;\mathrm{of}\;\mathrm{CF}\;\times \mathrm{water}\;\mathrm{content}\;\mathrm{of}\;\mathrm{CF}+\mathrm{water}\;\mathrm{addition}}{\mathrm{weight}\;\mathrm{of}\;\mathrm{WF}\;+\mathrm{weight}\;\mathrm{of}\;\mathrm{CF}+\mathrm{water}\;\mathrm{addition}}=45\%$$

### 2.3. Proximate Analysis

The proximate analysis for moisture, protein (N × 6.25), fat, crude fiber, ash, and starch content of flour samples was carried out according to the methods described by the AOAC [12].

### 2.4. Color Characteristics

The luminance (L*), red/green chromaticity (a*), and yellow/blue chromaticity (b*) of the flours and their dough samples were measured by NH300 colorimeter (Shenzhen 3nh Technology Co., Ltd., Shenzhen, China). 

### 2.5. Iodine Blue Value (IBV)

About 0.25 g of flour sample was dispersed in 50 mL of distilled water. The suspension was heated in a water bath at 65.5 °C for 1 min and then centrifuged for 10 min at 3000× *g*. An amount of 1 mL of supernatant was pipetted into a 50 mL colorimetric tube. A total of 1 mL of 0.02 mol/L standard solution of iodine was added, and the volume was diluted to the mark with water. A blank solution without sample treatment was used to set the spectrophotometer to zero, and the absorbance value at 650 nm was measured.

*IBV* = A × 54.2 + 5.

### 2.6. Functional Properties of Flours 

#### 2.6.1. Water Absorption Capacity (*WAC*, *g*/*g*) 

The *WAC* of samples was determined according to the method of Kaur [13] with slight modification. The flour sample (3.0 g, db, *W*_0_) was dispersed in distilled water (25 mL) and then placed into preweighed centrifuge tubes (*W_t_*). The dispersion was held for 30 min while stirring occasionally and then centrifuged for 20 min at 3000× *g*. The supernatant was decanted. The centrifuge tube was dried at 50 °C for 25 min in a hot air oven to remove the excess moisture, and the sample was reweighed (*W_s_*).

(1) $$WAC\;(g/g)=\frac{W_{s}-W_{t}-W_{0}}{W_{0}}$$

#### 2.6.2. The Water Solubility (*WS*, %) and Swelling Power (*SP*, %)

The *WS* and *SP* were determined according to the method of Wu [14] with slight modification. The flour sample (0.2 g, db, *W*_0_) was dispersed in distilled water (10 mL) and then heated with stirring for 30 min in a water bath at 80 °C. The prepared paste was cooled to room temperature and then centrifuged for 30 min at 3000× *g*. The supernatant was dried to constant weight at 105 °C, followed by weighing the dried solid (*W*_1_). The dried precipitate was also weighed (*W*_2_).

(2) $$WS\;(\%)=\frac{W_{1}}{W_{0}}\times 100$$

(3) $$SP\;(\%)=\frac{W_{2}}{W_{0}\times \left(1-WS\right)}\times 100$$

#### 2.6.3. Freeze–Thaw Stability (*FS*)

The flour sample was prepared into 6% suspensions with distilled water and was bathed in boiling water for 30 min. After cooling, about 20 g of starch paste (*W*_1_) was put into a preweighed centrifuge tube (*W*_2_) and frozen in a freezer at −20 °C for 24 h. After being thawed at room temperature for 6 h, it was centrifuged at 3000× *g* for 30 min, and then the supernatant was decanted. The centrifuge tube and precipitate were weighed (*W*_3_).

(4) $$FS\;(\%)=\frac{W_{2}-W_{3}}{W_{1}}\times 100$$

#### 2.6.4. Retrogradation

The flour sample was mixed with distilled water to form 1% emulsion, and it was stirred and heated in a boiling water bath for 20 min. The volume of starch paste was kept unchanged, and 15 mL of starch paste was transferred to a 25 mL tube after cooling to room temperature. After it was left to stand in 30 °C incubator for 48 h, the volume of supernatant (*V*_1_) and total (*V*) was recorded.

(5) $$\mathrm{Retrogradation}\;(\%)=\frac{V_{1}}{V}\times 100$$

### 2.7. Functional Properties of Dough

#### 2.7.1. Water Content

The water content of dough was determined by the AOAC [12] method.

#### 2.7.2. Sensory Evaluation

The sensory evaluation of dough was carried out for texture by a panel of ten trained assessors. Samples were randomly coded. Panelists were asked to describe the softness, elasticity, extensibility, and plasticity of the dough.

#### 2.7.3. Wet Gluten Content

The wet gluten content was determined using the hand-washing method using the AACC [15] method.

#### 2.7.4. Farinographic Characteristics

Water absorption, dough development time, dough stability time, and degree of softening, were measured by Micro-dough LAB farinograph (Perten, Stockholm, Sweden). The calculation results were corrected according to the water content of the dough. A certain amount of water was mixed with the sample to make the maximum consistency of the dough close to 500 FU, and the farinographic curve and data were obtained by testing. 

#### 2.7.5. Texture Analysis

The texture properties of the dough (including hardness, chewiness, elasticity, and cohesiveness) were measured by CT_3_ texture analyzer (Broofield, Middleboro, MA, USA). The dough was placed into a round mold with a diameter of 7 cm and a thickness of 2 cm. The settings were as follows: TA4/1000 cylindrical probe, TPA mode, test distance of 2.0 mm, test speed of 1.5 mm/s, and trigger force of 5.0 g. Each sample was determined six times, and the average value was taken.

#### 2.7.6. Extension Properties

The prepared dough was pressed into a 4 mm thick strip and then made into a dough strip with a length of 8 cm and a width of 1.5 cm. The extension properties of the dough (including resistance to extension, extensibility, extended area, and extension ratio) were measured by CT_3_ texture analyzer. The settings were as follows: A/KIE probe, tension mode, test speed of 2.0 mm/s, and trigger force of 10.0 g. Each sample was determined six times, and the average value was taken.

### 2.8. Statistics and Analysis Methods

Except for texture properties and extension properties, all other indexes were tested three times. The experimental data were statistically analyzed and plotted by Microsoft Excel 2016 and SPSS19.0 statistical data analytical software (SPSS Inc., Chicago, IL, USA). The analysis of variance and correlation analysis were performed by Duncan’s multiple comparison test (*p* < 0.05). 

## 3. Results and Discussion

### 3.1. Effects of CF Substitution Level on Properties of W-CF

#### 3.1.1. Proximate Analysis of W-CF

An increase in cassava flour substitution levels resulted in a gradual decrease in the moisture, protein, and fat content. This is attributed to the higher value of these compositions in CF compared with WF. The crude fiber, ash, and starch content of the composite flour increased gradually with increasing CF substitution levels. This was mainly due to the higher crude fiber, ash, and starch content in CF compared with WF, which is exceptionally cellulose-rich. The different substitution levels had a significant influence on the protein and crude fiber content of the composite flour (*p* < 0.05; Table 1).

#### 3.1.2. Color Characteristics of W-CF

Color is one of the most important factors affecting consumer acceptance [16]. The color characteristics of the W-CF and the control are shown in Table 2. The results show that the L* and a* values of the W-CF increased from 98.04 to 99.08 and 0.24 to 0.49, respectively, with increasing amounts of CF in the W-CF. The W-CF appeared more reddish and white, which is related to the natural color of CF. This is consistent with the results of Lagnika et al. [17]. The b* value of the W-CF decreased with an increasing CF substitution level. However, substituting up to 40% of WF with CF did not seem to cause major changes in the color. No significant difference existed between the values at substitution levels of 40% and 50%.

#### 3.1.3. Iodine Blue Value (*IBV*) of W-CF

**Figure 1:**
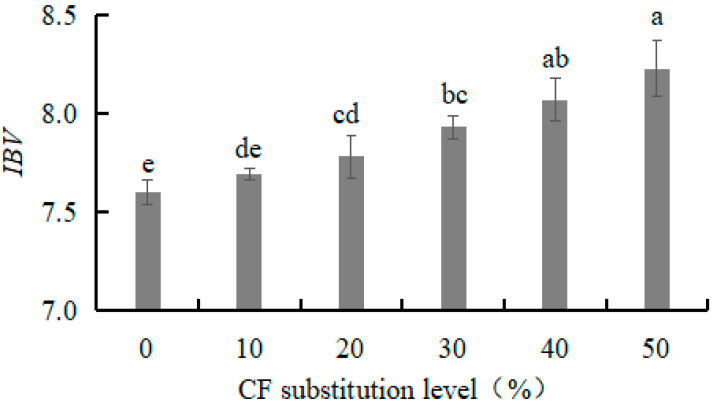
Effect of CF substitution level on *IBV* of W-CF. Note: Different lowercase letters indicate significant differences (*p* < 0.05) (the same below).

*IBV* reflects the free starch content in W-CF and can indicate the degree of cell damage. The *IBV* of the W-CF gradually increased from 7.69 to 8.23 with an increasing CF substitution level from 10% to 50% (Figure 1). The larger *IBV* indicated more free starch and a higher degree of cell damage. The results indicate that an increase in CF substitution level resulted in an increase in free starch content and damaged cells. This might be due to the differences in milling devices and conditions, resulting in different damaged starch contents. The high damaged starch content of CF may have been caused by the shear milling under dry conditions.

#### 3.1.4. Water Absorption Capacity (*WAC*) of W-CF

**Figure 2:**
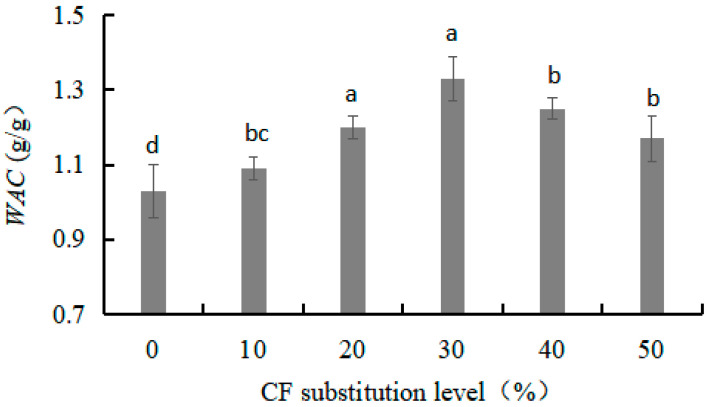
Effects of CF substitution level on *WAC* of W-CF.

The *WAC* reflects the ability of W-CF to bind and retain water. It was observed that an increase in CF substitution level resulted in a significant increase in *WAC* in the W-CF up to a maximum situated at the substitution level of 30%, after which they decreased (Figure 2). The high *WAC* of CF could be attributed to the presence of a higher amount of starch, especially free starch. The free starch granules had higher water absorption than that of the intact starch granules, which could help improve water retention [18]. However, CF has higher fiber content (1.89%) than WF (0.45%). The continuous increase in the substitution level enhanced the adsorption of water by the polar groups in the dietary fiber, leading to a slight decrease in the *WAC* of the W-CF [19,20]. The W-CF with high *WAC* may be useful in products that require good viscosity.

#### 3.1.5. Water Solubility (*WS*) and Swelling Power (*SP*) of W-CF

**Figure 3:**
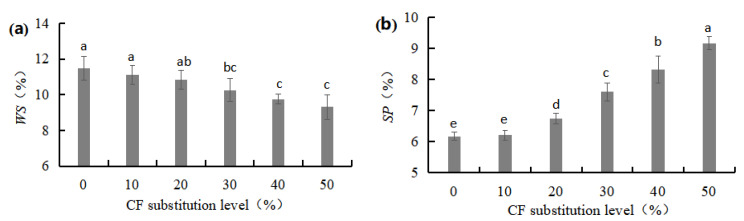
Effects of CF substitution level on the *WS* (**a**) and *SP* (**b**) of W-CF.

*WS* is the ability of W-CF to interact with a solution and dissolve. Figure 3a shows that there was a gradual decrease in the *WS* of the W-CF with an increase in CF substitution levels. The *WS* of the W-CF at a substitution level of 0~20% fluctuated minimally between 10.83% and 11.49%. When the substitution level was higher than 30%, the decline in the *WS* of the W-CF accelerated considerably, and the *WS* of the W-CF at substitution levels of 30~50% was 9.31~10.27%. In contrast to its solubility, the *SP* of the W-CF increased with an increase in CF substitution levels. The *SP* of the W-CF at substitution levels of 20~50% increased rapidly and was 9.40~48.78% higher than that of the control (Figure 3b). *SP* is the ability of W-CF granules to interact with water molecules and expand after water absorption. A higher *SP* of W-CF has been attributed to a weaker degree of intermolecular association and lower protein and fat content compared with WF [21,22].

#### 3.1.6. Freeze–Thaw Stability (*FS*) and Retrogradation of W-CF Gels

**Figure 4:**
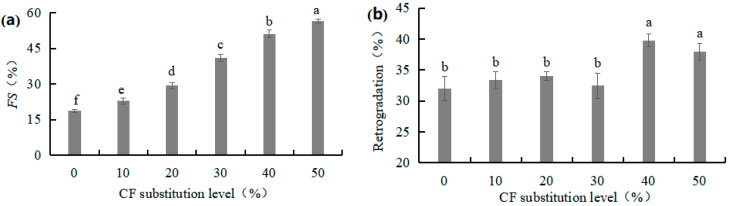
Effects of CF substitution level on the *FS* (**a**) and retrogradation (**b**) of W-CF.

*FS* is an index for evaluating the ability of W-CF to resist deterioration during freeze–thaw treatment. *FS* is expressed as the syneresis rate after freezing and thawing. A lower syneresis rate is related to better *FS*. The syneresis rate from the gelatinized W-CF pastes significantly increased with an increase in the CF substitution level, as shown in Figure 4a. A similar trend was observed by Lin et al. [23]. Higher syneresis on thawing indicated the structure of W-CF gels was easily disrupted by ice crystal formation, which is probably due to low intracellular and intermolecular hydrogen bonding.

Retrogradation reflects the gelling ability of the starch paste. Retrogradation is affected by numerous factors, including raw material processing method, composition, temperature, concentration, pH, starch molecular structure, molecular weight, and degree of polymerization [24]. There was no significant difference in retrogradation at substitution levels of 10~30%. The retrogradation of W-CF at substitution levels of 40~50% was significantly higher than that of the control and that at other substitution levels (*p* < 0.05; Figure 4b). This may be because the molecular structure of the starch paste rearranged to form a high-crystallinity structure, resulting in the retrogradation of the W-CF increasing. The retrogradation of the W-CF at a substitution level of 30% was the lowest (32.45%), possibly caused by different changes in the molecular structure of the starch paste when the substitution level reached a threshold.

#### 3.1.7. The Correlation between the CF Substitution Level and Properties of W-CF

Table 3 shows that the CF substitution level had a strong positive correlation with crude fiber, ash, and starch contents, L*, a*, *IBV*, *SP*, and *FS* (*p* < 0.01). It was significantly negatively correlated with the moisture, protein, and fat contents, b* and *WS* (*p* < 0.01). This indicated that the partial substitution of CF for WF had a significant influence on the proximate analysis and functional properties of W-CF, which might lead to changes in the W-CF physicochemical qualities, including rheological properties, pasting, and gelling.

### 3.2. Effects of CF Substitution Level on Functional Properties of Dough

#### 3.2.1. Water Content and Sensory Evaluation

The water content of the dough gradually decreased with an increase in substitution level. The water content of the dough significantly decreased when the substitution level exceeded 30%, and the difference in the water content of the dough between 40% and 50% was significant (*p* < 0.05; Table 4). Results from sensory evaluation indicated that adding CF had a significant effect on the hardness, elasticity, and extensibility of the dough. The addition of CF to the dough had the significant effect of increasing hardness and reducing the extensibility. The texture of the dough was acceptable. As the level of CF replacement increased, the dough made with CF was stiff and difficult to sheet and mold. Further replacement resulted in decreased handing properties. The CF dough has no gluten, and the gluten content of the W-CF dough decreased after mixing with WF. Additionally, the adsorption of water by polar groups such as dietary fiber in CF were continuously enhanced [19,20].

#### 3.2.2. Wet Gluten Content of Dough

**Figure 5:**
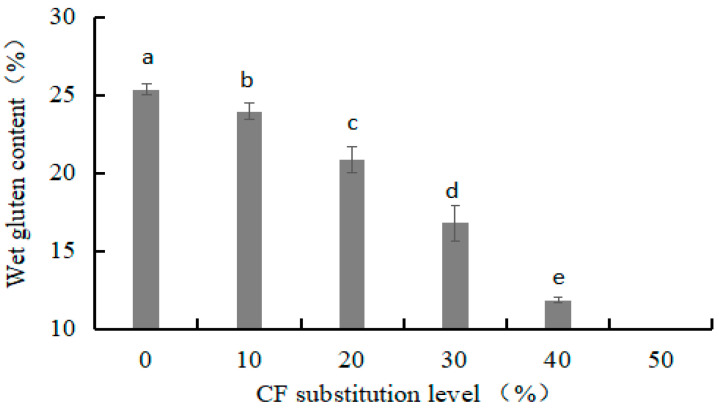
Effects of CF substitution level on wet gluten content of dough.

Gliadin and glutenin are the main components of wet gluten. These are important indices for evaluating WF and predicting the quality of processed products [25]. A decrease in wet gluten content will significantly affect the rheological, gas retention, and texture properties of dough [26]. As shown in Figure 5, it was expected that an increase in CF substitution level would result in a continual decrease in the wet gluten content of the dough (*p* < 0.05). At 50% replacement, the wet gluten content could not be effectively measured because an obvious gluten film was not observed. These results were consistent with the findings of Lagnika et al. [17] and Oladunmoye et al. [27]. This is because the CF did not contain gluten protein. Thus, the addition of CF reduced the proportion of gluten protein in the dough.

#### 3.2.3. Farinographic Characteristics of Dough

##### Water Absorption

Water absorption is mainly dependent on the ability of W-CF gluten and damaged starch to bind to water. Additionally, water absorption is related to the starch, protein, dietary fiber, and polysaccharides in W-CF, as well as starch granule properties, gelatinization degree, and other factors [28]. As expected, the water absorption of the W-CF gradually increased with an increase in CF substitution level, reaching 64.40% at a substitution level of 50%, which was 11.42% higher than that of the control (57.80%; Table 5). The difference in the W-CF water absorption among the different substitution levels was significant (*p* < 0.05). The main reasons for this were as follows: (i) CF contained a large quantity of starch, which has higher and faster water-binding ability than gluten [29]. (ii) CF had a smaller granule size and loose structure, making it was easier to bind to water. The damage-free starch in CF was broken and disintegrated, and the internal structure was exposed, allowing water molecules to enter [30]. (iii) CF is rich in dietary fiber, which has strong water absorption and water-holding capacities [31]. This was consistent with the results of Khoshgozaran-Abras et al. [32].

##### Dough Development Time 

The dough development time reflects the formation speed of the gluten network structure and water-binding ability of flour. The development time of the dough tended to decrease for CF contents from 10% to 40%, followed by a significant increase at 50% CF content in the W-CF (*p* < 0.05; Table 5). The decrease in dough formation time could be attributed to the dilution of gluten protein [33]. This result is consistent with that of mung bean–wheat [34] and potato–wheat composite flour [35]. At 50% replacement, the rise in dough formation time may be related to the impairment of the gluten network structure.

##### Stability Time

Stability time is the ability of the internal structure of the dough to withstand external mechanical forces and is an important index for evaluating the internal quality of dough. In general, longer stability time is related to greater gluten strength and better machinability. Table 5 indicates the stability time of the dough tended to decrease for CF contents from 10% to 40%, followed by a slight increase at 50% CF content in the W-CF. Similar results were reported by Wu et al. [36] for brown rice–wheat dough. The decrease in the stability time of the dough can mainly be attributed to the decrease in protein content, resulting in rapid development and hydration [37].

##### Softening Degree

The softening degree is the degree of damage to the gluten network structure of the dough during mixing and kneading. As shown in Table 5, regardless of the substitution level, the softening degree of the dough was higher than that of the control. The softening degree increased to the maximum of 54.00 mN·m at 20% replacement, followed by a slight decrease at 30~50% CF content in the W-CF. The increased softening degree of the dough may be related to the dilution of gluten and interaction between the fiber and gluten [36].

#### 3.2.4. Textural Properties of Dough

A texture analyzer can objectively and accurately evaluate the textural properties of dough. The result indicates that the dough made with CF showed an increase in hardness and chewiness, and the difference among different substitution levels was significant (*p* < 0.05; Table 6). The hardness increased significantly when the substitution level exceeded 30%, and when substitution level was 50%, it reached 5376.33 g, which was 2.32 times that of the control. The chewiness significantly increased when the substitution level exceeded 40%, and at a substitution level of 50%, it reached 60.22 mJ, which was 2.70 times that of the control. The softness worsened, because gluten was not present in the CF and therefore not conducive to gluten network formation. Additionally, the CF was rich in dietary fiber and had strong water absorption properties.

Table 6 shows that the elasticity and cohesiveness of the dough tended to increase for CF contents from 10% to 30%. This change in trend was consistent with *WAC*. Thus, the partial replacement of WF with CF would yield desirable textural properties of dough. However, when substitution levels exceeded 30%, the elasticity and cohesiveness of the dough tended to decrease. This is consistent with the dough properties in Table 4, and it is possibly caused by the increased substitution level forcing the starch in the W-CF to compete with gluten for water and causing some of the gluten molecules to absorb insufficient water so that a gluten network could not be formed, resulting in a weakened dough structure and affecting its elasticity [38]. This relates to other components, starch types, and CF characteristics, as well as their interaction with starch and protein in WF [39].

#### 3.2.5. Extension Properties of Dough

As shown in Table 7, the resistance to extension, extensibility, and the extended area of the W-CF dough at all substitution levels were significantly lower than those of the WF dough, and the difference among different substitution levels was significant (*p* < 0.05). There was no significant difference in the extension ratio of dough at different substitution levels. The extension properties of dough are primarily governed by the polymeric network of gluten proteins. Thus, the results indicate that using 10~50% CF to replace WF has a significant and unfavorable influence on the extension properties of dough, especially substitution levels exceeding 30%. A higher substitution level is related to worse the gluten strength and extensibility of dough, which is consistent with the results of Wang [35] on potato–wheat composite flour.

#### 3.2.6. Correlation between CF Substitution Level and Dough Properties

Pearson’s correlation analysis showed that the CF substitution level was significantly and positively correlated to the water absorption, hardness, and chewiness of the W-CF dough (*p* < 0.01; Table 8). An inverse correlation was found between the substitution level of CF with water content, wet gluten, resistance to extension, extensibility, and extended area (*p* < 0.01). The result indicates that a higher CF substitution level resulted in higher water absorption, hardness, and chewiness, as well as lower water content, wet gluten, resistance to extension, extensibility, stability time, and cohesiveness of the dough. However, with the CF substitution level increasing, the gluten strength weakened, the ability of the internal structure to withstand external mechanical forces worsened, the dough hardened, and extensibility, elasticity, and operability worsened.

## 4. Conclusions

In this study, the influence of the partial substitution of 0~50% WF by CF on the functional properties of composite flours and their doughs was investigated. The results indicate that the substitution levels of CF had a significant influence on the proximate analysis and functional properties of W-CF and dough. Thus, this study could be of significance in choosing CF substitution levels for different products. After replacing WF with a certain proportion of CF, there was an effective improvement in the crude fiber and starch content, whiteness, and *SP* of the W-CF. The water absorption, elasticity, and cohesiveness of the W-CF gradually increased for CF contents from 10% to 30%, which may be useful in products that require unique properties. On other hand, the addition of CF to dough has the significant effect of increasing hardness and reducing extensibility. The functional properties of the dough declined obviously when the substitution level exceeded 30%. Thus, 30% replacement is the best level for preparing baked products. This study will provide an important theoretical basis for the processing and comprehensive utilization of CF. Moreover, the nutritional enhancement of W-CF and dough should be considered.

## Figures and Tables

**Table 1:** The basic components of W-CF.

CF Substitution Level (%)	Moisture (%)	Protein (%)	Fat (%)	Crude Fiber (%)	Ash (%)	Starch (%)
0	12.53 ± 0.24 ^a^	8.49 ± 0.10 ^a^	1.72 ± 0.11 ^a^	0.45 ± 0.07 ^g^	0.73 ± 0.08 ^e^	70.27 ± 1.53 ^d^
10	12.19 ± 0.17 ^b^	7.92 ± 0.11 ^b^	1.63 ± 0.11 ^ab^	0.58 ± 0.02 ^f^	0.83 ± 0.06 ^de^	70.71 ± 1.02 ^cd^
20	11.83 ± 0.14 ^c^	7.30 ± 0.07 ^c^	1.54 ± 0.15 ^abc^	0.77 ± 0.04 ^e^	0.95 ± 0.05 ^d^	71.41 ± 1.02 ^bcd^
30	11.63 ± 0.12 ^c^	6.68 ± 0.11 ^d^	1.48 ± 0.11 ^bc^	0.88 ± 0.05 ^d^	1.10 ± 0.07 ^c^	71.97 ± 0.46 ^bcd^
40	11.29 ± 0.17 ^d^	6.03 ± 0.08 ^e^	1.38 ± 0.01 ^cd^	1.05 ± 0.03 ^c^	1.20 ± 0.04 ^bc^	72.40 ± 0.38 ^bc^
50	11.07 ± 0.03 ^d^	5.35 ± 0.13 ^f^	1.26 ± 0.16 ^d^	1.18 ± 0.08 ^b^	1.32 ± 0.11 ^b^	72.95 ± 1.27 ^b^
100	9.58 ± 0.15 ^e^	2.21 ± 0.15 ^g^	1.02 ± 0.09 ^e^	1.89 ± 0.09 ^a^	1.90 ± 0.07 ^a^	75.01 ± 0.78 ^a^

Note: Data were mean ± SD; Different superscript letters along the column are significantly different (*p* < 0.05) (the same below).

**Table 2:** Effects of CF substitution level on color characteristics of W-CF.

CF Substitution Level (%)	Colorimetric Value of W-CF		
	L*	a*	b*
0	98.04 ± 0.22 ^d^	0.24 ± 0.04 ^d^	4.52 ± 0.15 ^a^
10	98.48 ± 0.15 ^c^	0.34 ± 0.03 ^c^	4.42 ± 0.08 ^a^
20	98.54 ± 0.14 ^c^	0.39 ± 0.02 ^b^	4.26 ± 0.06 ^b^
30	98.73 ± 0.10 ^b^	0.42 ± 0.03 ^b^	4.06 ± 0.08 ^c^
40	98.95 ± 0.08 ^a^	0.46 ± 0.03 ^a^	3.84 ± 0.14 ^d^
50	99.08 ± 0.10 ^a^	0.49 ± 0.02 ^a^	3.72 ± 0.05 ^d^

**Table 3:** Correlation between CF substitution level and the properties of W-CF.

Indexes	CF Substitution Level	Moisture	Protein	Fat	Fiber	Ash	Starch	L*	a*	b*	*IBV*	*WAC*	*WS*	*SP*	*FS*	Retrogradation
Moisture	−1.00 **	1														
Protein	−1.00 **	0.99 **	1													
Fat	−1.00 **	0.99 **	1.00 **	1												
Fiber	1.00 **	−1.00 **	−1.00 **	−0.99 **	1											
Ash	1.00 **	−0.99 **	−1.00 **	−0.99 **	1.00 **	1										
Starch	1.00 **	−1.00 **	−1.00 **	−0.99 **	1.00 **	1.00 **	1									
L*	0.98 **	−0.98 **	−0.97 **	−0.97 **	0.97 **	0.97 **	0.97 **	1								
a*	0.97 **	−0.98 **	−0.96 **	−0.96 **	0.97 **	0.96 **	0.97 **	0.99 **	1							
b*	−0.99 **	0.99 **	1.00 **	0.99 **	−0.99 **	−1.00 **	−0.99 **	−0.96 **	−0.95 **	1						
*IBV*	0.99 **	−0.98 **	−1.00 **	−0.99 **	0.99 **	0.99 **	0.99 **	0.96 **	0.94 **	−0.99 **	1					
*WAC*	0.65	−0.67	−0.63	−0.59	0.65	0.66	0.68	0.66	0.72	−0.63	0.58	1				
*WS*	−1.00 **	0.99 **	1.00 **	0.99 **	−0.99 **	−1.00 **	−0.99 **	−0.97 **	−0.95 **	1.00 **	−1.00 **	−0.6	1			
*SP*	0.98 **	−0.96 **	−0.98 **	−0.98 **	0.97 **	0.98 **	0.97 **	0.92 **	0.90 **	−0.99 **	0.99 **	0.54	−0.99 **	1		
*FS*	0.99 **	−0.98 **	−0.99 **	−0.98 **	0.99 **	0.99 **	0.99 **	0.95 **	0.94 **	−1.00 **	0.99 **	0.63	−1.00 **	0.99 **	1	
Retrogradation	0.80 *	−0.81 *	−0.80 *	−0.81 *	0.81 *	0.78 *	0.77 *	0.80 *	0.76 *	−0.83 *	0.81 *	0.29	−0.82 *	0.80 *	0.82 *	1

Note: “*” and “**”, respectively, indicate that the correlation reached significant level (*p* < 0.05) and extremely significant level (*p* < 0.01).

**Table 4:** Effects of CF substitution level on water content and description of dough properties.

CF Substitution Level (%)	Water Content (%)	Description of Dough Properties
0	44.48 ± 0.61 ^a^	Too soft, good extensibility and plasticity, very good elasticity, easy to sheet and mold.
10	43.91 ± 0.19 ^ab^	Very soft, very good extensibility, malleability, and elasticity, does not stick to hand, easier to sheet and mold.
20	43.10 ± 0.10 ^bc^	Soft, good extensibility, malleability, and elasticity, does not stick to hand, easier to sheet and mold.
30	42.57 ± 0.64 ^c^	Softer, good extensibility, elasticity, and plasticity, easy to sheet and mold.
40	41.34 ± 0.69 ^d^	Poor softness, poor extensibility and plasticity, general elasticity, sticky, not easy to sheet or mold.
50	39.64 ± 0.51 ^e^	Hard, poor extensibility and plasticity, basically inelastic, very sticky, difficult to sheet and mold.

**Table 5:** Effects of CF substitution level on the farinographic characteristics of dough.

CF Substitution Level (%)	Farinographic Characteristics of Dough			
	Water Absorption (%)	Development Time (min)	Stability Time (min)	Degree of Softening (mN·m)
0	57.80 ± 0.00 ^f^	1.63 ± 0.06 ^a^	1.97 ± 0.06 ^a^	44.67 ± 1.15 ^c^
10	59.50 ± 0.00 ^e^	0.97 ± 0.06 ^c^	1.70 ± 0.06 ^b^	45.67 ± 2.08 ^c^
20	60.80 ± 0.00 ^d^	0.80 ± 0.00 ^d^	0.93 ± 0.06 ^c^	54.00 ± 0.00 ^a^
30	62.00 ± 0.20 ^c^	0.83 ± 0.06 ^cd^	0.67 ± 0.06 ^d^	52.67 ± 2.52 ^ab^
40	63.77 ± 0.25 ^b^	0.90 ± 0.10 ^cd^	0.67 ± 0.06 ^d^	52.67 ± 2.31 ^ab^
50	64.40 ± 0.17 ^a^	1.13 ± 0.12 ^b^	0.90 ± 0.06 ^c^	50.00 ± 1.00 ^b^

**Table 6:** Effects of CF substitution level on texture properties of dough.

CF Substitution Level (%)	Texture Properties of Dough			
	Hardness (g)	Chewiness (mJ)	Elasticity (mm)	Cohesiveness
0	2320.33 ± 140.68 ^f^	22.27 ± 2.84 ^f^	1.34 ± 0.11 ^c^	0.63 ± 0.04 ^b^
10	2731.67 ± 230.82 ^e^	27.07 ± 4.51 ^e^	1.46 ± 0.10 ^b^	0.66 ± 0.04 ^b^
20	3214.67 ± 433.18 ^d^	33.77 ± 3.09 ^d^	1.63 ± 0.06 ^a^	0.71 ± 0.05 ^a^
30	3588.17 ± 372.41 ^c^	39.82 ± 2.37 ^c^	1.27 ± 0.08 ^cd^	0.61 ± 0.03 ^b^
40	4683.83 ± 268.69 ^b^	45.73 ± 2.64 ^b^	1.17 ± 0.08 ^d^	0.46 ± 0.04 ^c^
50	5376.33 ± 263.94 ^a^	60.22 ± 3.52 ^a^	0.99 ± 0.09 ^e^	0.43 ± 0.04 ^c^

**Table 7:** Effects of CF substitution level on the extension properties of dough.

CF Substitution Level (%)	Extension Properties of Dough			
	Resistance to Extension (g)	Extensibility (mm)	Extended Area (g·m)	Extension Ratio (g/mm)
0	32.50 ± 2.59 ^a^	43.80 ± 2.64 ^a^	1419.37 ± 82.58 ^a^	0.75 ± 0.10 ^a^
10	28.17 ± 1.17 ^b^	39.32 ± 1.87 ^b^	1107.21 ± 64.26 ^b^	0.72 ± 0.05 ^a^
20	23.83 ± 1.47 ^c^	30.58 ± 2.07 ^c^	726.53 ± 23.26 ^c^	0.78 ± 0.10 ^a^
30	17.33 ± 1.21 ^d^	23.99 ± 3.13 ^d^	413.51 ± 36.70 ^d^	0.74 ± 0.13 ^a^
40	13.83 ± 1.17 ^e^	20.00 ± 1.49 ^e^	276.97 ± 34.09 ^e^	0.69 ± 0.07 ^a^
50	10.33 ± 0.82 ^f^	15.34 ± 1.09 ^f^	158.94 ± 21.58 ^f^	0.67 ± 0.05 ^a^

**Table 8:** Correlation between CF substitution level and dough properties.

Indexes	Water Content	Wet Gluten	Water Absorption	Development time	Stability Time	Degree of Softening	Hardness	Chewiness	Elasticity	Cohesiveness	Resistance to Extension	Extensibility	Extended Area	Extension Ratio
CF Substitution level	−0.98 **	−0.98 **	0.99 **	−0.46	−0.84 *	0.63	0.98 **	0.98 **	−0.71	−0.81 *	−1.00 **	−0.99 **	−0.98 **	−0.7
Water content	1	0.99 **	−0.96 **	0.3	0.71	−0.49	−0.99 **	−1.00 **	0.77 *	0.86 *	0.96 **	0.95 **	0.91 **	0.76 *
Wet gluten		1	−0.98 **	0.59	0.89 **	−0.75	−0.99 **	−0.99 **	0.6	0.78 *	0.98 **	0.97 **	0.95 **	0.55
Water absorption			1	−0.52	−0.86 *	0.66	0.97 **	0.96 **	−0.67	−0.79 *	−0.99 **	−0.99 **	−0.98 **	−0.69
Development time				1	0.75	−0.77 *	−0.33	−0.33	−0.2	−0.06	0.48	0.5	0.6	−0.02
Stability time					1	−0.93 **	−0.74	−0.74	0.33	0.44	0.86 *	0.89 **	0.93 **	0.26
Degree of softening						1	0.52	0.52	0	−0.15	−0.65	−0.71	−0.76 *	0.07
Hardness							1	0.98 **	−0.77 *	−0.88 **	−0.97 **	−0.96 **	−0.93 **	−0.78 *
Chewiness								1	−0.77 *	−0.83 *	−0.97 **	−0.96 **	−0.93 **	−0.74
Elasticity									1	0.94 **	0.72	0.67	0.6	0.88 **
Cohesiveness										1	0.80 *	0.76 *	0.7	0.92 **
Resistance to extension											1	1.00 **	0.99 **	0.68
Extensibility												1	0.99 **	0.62
Extended area													1	0.57

Note: “*” and “**”, respectively, indicate that the correlation reached significant level (*p* < 0.05) and extremely significant level (*p* < 0.01)

## Data Availability

The data presented in this study are available on request from the corresponding author or first author.
